# Neuregulin-1β Mitigates Doxorubicin-Induced Cardiotoxicity via *Serping1* in Cardiac Fibroblasts

**DOI:** 10.3390/ijms27104616

**Published:** 2026-05-21

**Authors:** Parisa Aghagolzadeh, Lifen Xu, Philipp Klinger, Christian Morandi, Lilia Maryse Lépine, Lukas Minder, Pieter-Jan Guns, Matthias Bosman, Marie-May Coissieux, Thierry Pedrazzini, Gabriela Kania, Marijke Brink

**Affiliations:** 1 Cardiobiology, Department of Biomedicine, University of Basel and University Hospital Basel, 4031 Basel, Switzerland; 2 Center of Experimental Rheumatology, Department of Rheumatology, University Hospital Zurich, University of Zurich, 8091 Zurich, Switzerlandgabriela.kania@uzh.ch (G.K.); 3 Laboratory of Physiopharmacology, Faculty of Medicine and Health Sciences, Faculty of Pharmaceutical, Biomedical and Veterinary Sciences, Campus Drie Eiken, University of Antwerp, 2610 Antwerp, Belgium; 4 Laboratory of Tumor Heterogeneity, Metastasis and Resistance, Department of Biomedicine, University of Basel and University Hospital Basel, 4031 Basel, Switzerland; 5 School of Cardiovascular and Metabolic Medicine and Sciences, MRC/BHF Centre of Research Excellence in Advanced Cardiac Therapies, King’s College London, London WC2R 2LS, UK

**Keywords:** cardiac remodeling, anthracycline cardiotoxicity, cardiac fibroblasts, cardioprotection

## Abstract

Anthracyclines such as doxorubicin (DOX) are widely used in cancer treatment, but their benefits are offset by dose-related cardiotoxicity. Neuregulin-1β (NRG1) has been studied as a cardioprotective factor, yet its mechanisms during DOX treatment, particularly in the presence of cancer, are not well understood. This study evaluated daily recombinant NRG1 co-administered with DOX in 4T1-tumor-bearing female BALB/c mice. The mice were randomized to saline, DOX (3 mg/kg i.p. on days 0, 3, 6, 9; cumulatively 12 mg/kg) or DOX + NRG1 (20 µg/kg i.p. daily, starting one day before DOX). Body weight and tumor growth were monitored throughout treatment. Cardiac structure and function were assessed by transthoracic echocardiography at baseline and before sacrifice. Mechanistic studies included left ventricular proteomics and single-cell RNA-seq. We also used human 3D cardiac microtissues and 2D primary cardiac fibroblast-enriched cultures under defined experimental conditions, with targeted fibroblast gene perturbations. We found that early DOX exposure induced systolic dysfunction and pathological remodeling, while daily NRG1 preserved the ejection fraction and attenuated structural changes without impairing anti-tumor efficacy. Proteomic analysis identified *Serping1* as one of the most strongly upregulated proteins soon after DOX exposure, an effect that was reversed by NRG1. Notably, *Serping1* has not previously been implicated in anthracycline cardiotoxicity or NRG1-mediated protection. Single-cell RNA sequencing localized *Serping1* expression to cardiac fibroblasts. Mechanistically, we found that *Serping1* modulation was associated with altered *Igfbp5* processing and fibroblast survival under DOX-induced stress; its suppression by NRG1 was linked to reduced fibroblast apoptosis and a shift toward a pro-survival-associated state. In human cardiac microtissues, NRG1 treatment or fibroblast-specific *Serping1* knockdown accelerated cardiomyocyte contraction dynamics. These changes occurred without an increase in apoptosis and point to a paracrine effect of fibroblasts on cardiomyocyte function. Additionally, scRNA-seq revealed an *Erbb4+* fibroblast subpopulation associated with early pro-fibrotic activation that expanded after DOX but was reduced by NRG1. Taken together, NRG1 preserved cardiac function during anthracycline treatment while maintaining anti-tumor efficacy. Our data identify fibroblast-associated signaling, particularly through *Serping1*, as a potential contributor to the early protective effects of NRG1. These findings add a new dimension to the understanding of NRG1 cardioprotection and suggest that fibroblast–myocyte interactions may contribute to the early cardiac response to DOX.

## 1. Introduction

Anthracyclines such as doxorubicin (DOX) remain among the most effective chemotherapeutic agents for breast cancer and a range of other malignancies, including colon, esophageal and lung cancers, leukemia and osteosarcoma. However, doxorubicin’s clinical utility is limited by cumulative and often irreversible cardiotoxicity [1,2,3,4]. Cardiotoxic complications may be acute (observed within days), delayed (within weeks or months) or manifest long-term (4–20 years) after DOX treatment, with reported incidences of irreversible heart failure ranging from 0.4 to 41% [4,5,6,7]. Multiple mechanisms have been proposed to explain this toxicity, including reactive oxygen species (ROS) generation, mitochondrial damage, topoisomerase II–mediated DNA injury and maladaptive activation of innate immune responses within the myocardium [8,9]. While these processes primarily affect cardiomyocytes, it is increasingly evident that non-myocyte populations, such as cardiac fibroblasts, play an active role in the remodeling response to anthracycline exposure [4,10,11].

Neuregulin-1β (NRG1), a member of the epidermal growth factor family, has emerged as a promising cardioprotective factor. NRG1 is secreted by endothelial cells in response to stress and acts through ErbB receptor tyrosine kinases on cardiomyocytes and other cell types in the heart. In preclinical models, NRG1 has been shown to enhance cardiomyocyte survival, improve contractile performance and attenuate pathological remodeling [12,13,14]. Early clinical trials in patients with chronic heart failure demonstrated that recombinant human NRG1 improved the LV ejection fraction and symptoms, supporting its translational potential [15,16]. However, its effects in the context of anthracycline therapy, where cardioprotection must be achieved without impairing anti-tumor efficacy, remain incompletely defined.

Cardiac fibroblasts, which account for the majority of non-myocyte cells in the heart, are now recognized as dynamic regulators of myocardial structure and function. They influence tissue remodeling, immune cell recruitment and even cardiomyocyte contractility through paracrine signaling [17,18,19]. Recent single-cell analyses have revealed fibroblast heterogeneity, including transitional and activated states that may critically shape cardiac injury responses [20,21,22].

Previous studies have shown that NRG1 can attenuate doxorubicin-induced cardiotoxicity through cardiomyocyte-centered mechanisms, including reduced oxidative stress and the preservation of excitation–contraction coupling, maintenance of cardiac troponins, ErbB-dependent survival signaling and, more recently, YAP-mediated inhibition of senescence [23,24,25]. However, whether NRG1 provides cardioprotection in the presence of an active tumor burden without compromising doxorubicin’s anti-tumor efficacy, and whether non-cardiomyocyte populations contribute to this response, is less well defined.

In the present study, we employed a tumor-bearing breast cancer model to examine whether NRG1 mitigates early DOX-induced cardiac dysfunction while preserving its anti-cancer efficacy. Using integrated proteomic and single-cell transcriptomic approaches, we identified *Serping1* as a fibroblast-enriched and NRG1-responsive protein. *Serping1* links to *Igf1*/*Igfbp5* survival signaling and exerts both autocrine effects within fibroblasts and paracrine effects on cardiomyocyte contractility. Furthermore, we demonstrate that NRG1 selectively reduces an *Erbb4+* fibroblast subpopulation associated with early pro-fibrotic activation. By focusing on fibroblast-specific pathways, our work highlights a novel dimension of NRG1-mediated cardioprotection and provides mechanistic insight into how protective signaling can be targeted for therapeutic intervention in the context of anthracycline therapy.

## 2. Results

### 2.1. NRG1 Reduces DOX-Induced Cardiotoxicity Without Impairing the Growth-Inhibiting Properties of DOX

**Figure 1:**
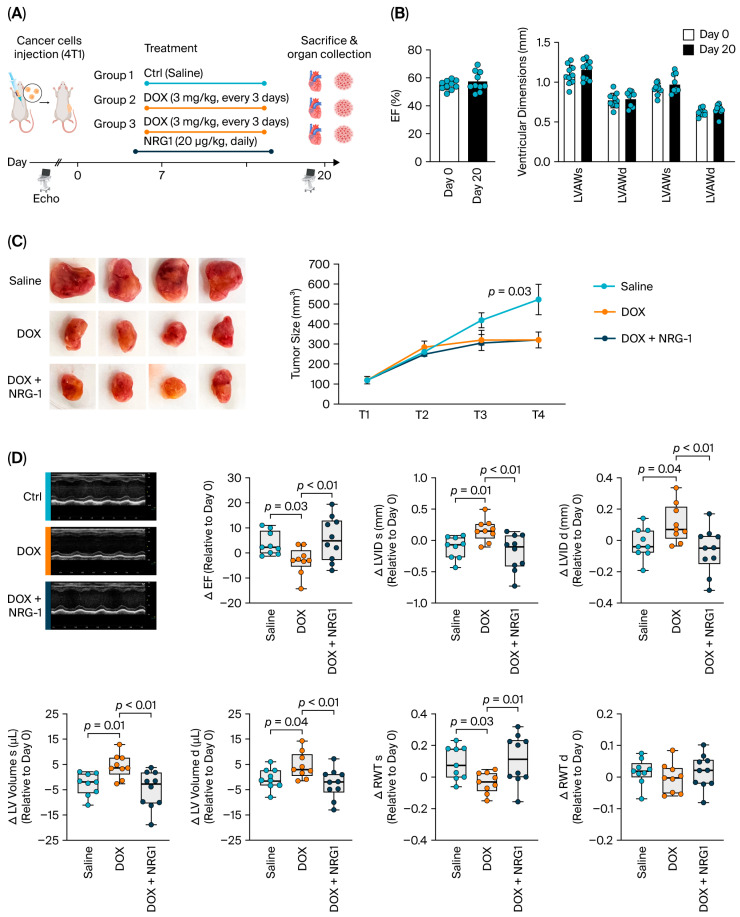
The cardiotoxicity induced by DOX is reduced by NRG1, while its anti-cancer properties are maintained. (**A**) Schematic overview of mouse experiments. Tumor growth was monitored in mice injected with 4T1 breast cancer cells. Mice were treated with a cumulative dose of 12 mg/kg doxorubicin (DOX) or saline. NRG1 treatment was initiated before DOX administration and continued daily. (**B**) Echocardiographic analysis showed preserved ejection fraction (EF) and ventricular dimensions in tumor-bearing mice. Assessments were performed at baseline and 20 days after the injection of 4T1 breast cancer cells. (**C**) Tumor size was monitored at different time points: T1 (7 days post-4T1 injection), T2, T3 and T4 (every 3–4 days thereafter). *p* values were calculated by two-way ANOVA. Representative images of tumor size are shown (**left**). (**D**) Echocardiographic assessment of cardiac dimension and function, with representative M-mode images of the left ventricle in untreated, DOX-treated, and DOX + NRG1-treated mice on day 20. Data are mean ± SD and exact *p* values are shown. Unless otherwise noted, group differences (Saline, DOX, DOX + NRG1) were analyzed by one-way ANOVA, followed by Fisher’s LSD for comparisons (DOX vs. Saline, DOX + NRG1 vs. DOX, and DOX + NRG1 vs. Saline) when the overall ANOVA result was significant (*p* < 0.05). EF, ejection fraction; LVID, left ventricular internal diameter; LV volume, left ventricular volume; d, end diastole; s, end systole; RWT, relative wall thickness; RWT = (2 × LVPW)/LVID. *N* = 9–10 mice per group (biological replicates) for all graphs in panels (**B**–**D**); each symbol represents one biological replicate (individual mouse).

We established a DOX-treated, tumor-bearing mouse model to simultaneously assess NRG1′s cardioprotective effects and tumor growth. We used BALB/c mice injected with 4T1 cells to closely mimic human breast cancer. A total of 1 × 10^5^ 4T1 cells were implanted into the mammary fat pads of 8–11-week-old BALB/c mice (Figure 1A). In a first experiment, we investigated the effect of the tumor itself on cardiac function. Our echocardiographic analysis showed that 20 days after 4T1 cell injection, the ejection fraction (EF) was preserved (Figure 1B). Ventricular dimensions, including left ventricular anterior wall (LVAW) and posterior wall (LVPW) thickness at the end of diastole and systole, were also unchanged (Figure 1B). These findings indicate that, at this early time point, the presence and growth of tumor cells did not alter cardiac function.

Next, 4T1 cells were implanted into the mammary fat pads of all mice to obtain three experimental groups. After 7 days, when the mean tumor size reached approximately 100 mm^3^, the mice were randomized for treatment with saline (control), DOX and DOX + NRG1. DOX (3 mg/kg) was administered intraperitoneally (i.p.) four times on days 0, 3, 6 and 9. NRG1 or saline was administered via i.p. injection starting one day before the first DOX injection and continued daily until the end of the experiment (Figure 1A).

Our results demonstrated that DOX treatment led to a significant reduction in body weight and that NRG1 was unable to prevent this weight loss (Supplementary Figure S1A). DOX effectively reduced tumor size compared to the control group. Importantly, NRG1 did not interfere with these growth-inhibiting effects of DOX, as both the DOX and DOX + NRG1 groups showed similar reductions in tumor size (Figure 1C).

Echocardiography was performed at baseline (prior to tumor cell injection) and prior to sacrifice. We calculated the change (Δ) in cardiac dimensions and function between the two time points for the three experimental groups (Figure 1D and Supplementary Table S1). DOX treatment led to a significant reduction in ejection fraction (EF). This reduction was attenuated by NRG1 co-treatment (*p* < 0.01 vs. DOX), which restored EF to levels comparable to those of the control group. DOX administration induced pathological changes, as shown by increased left ventricular internal diameters (LVIDs) and increased left ventricular volumes (LV Vol) at both end-diastole and end-systole. These structural changes were mitigated by NRG1 co-treatment.

Notably, the DOX-treated mice exhibited a significant reduction in the ratio of left ventricle weight to tibia length, indicating cardiac atrophy (Supplementary Figure S1B). NRG1 co-treatment prevented this cardiac weight loss, further supporting its protective effect on myocardial integrity. DOX treatment reduced relative wall thickness (RWT) at end-systole (*p* = 0.03 vs. saline), an effect prevented by NRG1 treatment (*p* = 0.01 vs. DOX). In contrast, no significant changes were observed in diastolic RWT among groups (Figure 1D)

In summary, these results suggest that NRG1 protects against early DOX-induced cardiotoxicity without interfering with the tumor-growth-inhibiting effects of DOX.

### 2.2. Proteomic Profiling Reveals Serping1 as a NRG1-Modulated Target in Cardiac Fibroblasts After DOX Treatment

**Figure 2:**
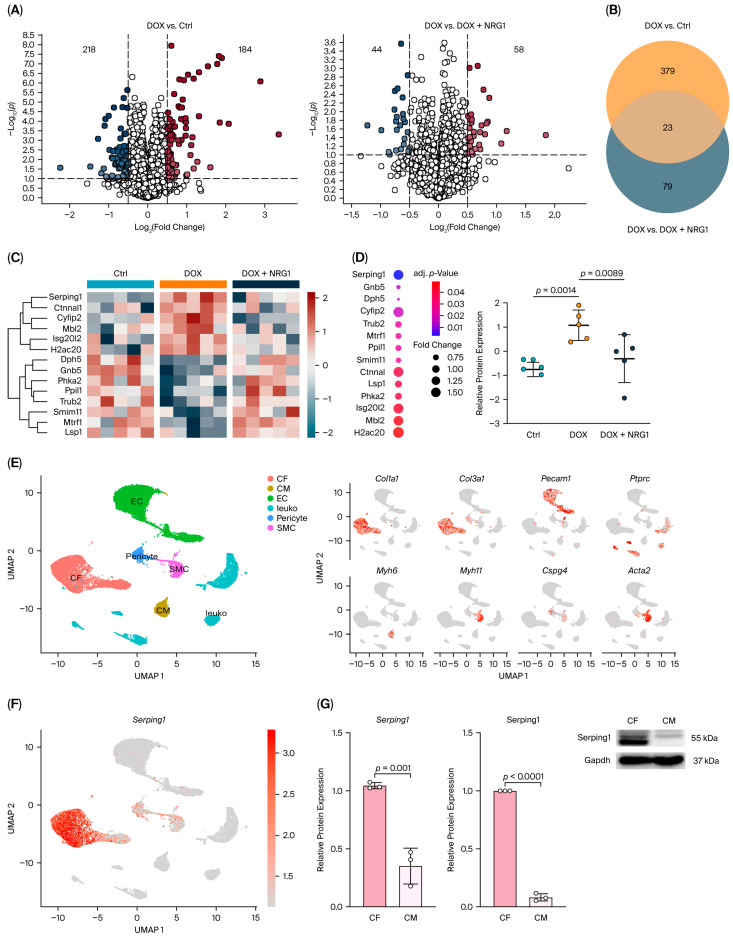
Proteomic profiling reveals *Serping1* as an NRG1-modulated target in cardiac fibroblasts after DOX treatment. (**A**) Volcano plots showing differentially expressed proteins in DOX-treated versus Ctrl hearts (**left**) and DOX versus DOX + NRG1-treated hearts (**right**). Numbers indicate significantly upregulated (red) and downregulated (blue) proteins (complete list provided in Supplementary Table S1). Panel A is based on proteomic data from *N* = 5 mice per group (biological replicates). (**B**) Venn diagram showing the overlap of differentially expressed proteins between DOX vs. Ctrl and DOX vs. DOX + NRG1 comparisons, identifying 23 common proteins. (**C**) Heatmap of 14 overlapping proteins (a subset of the 23 in Figure 2B) that change in opposite directions between DOX and DOX + NRG1, with DOX + NRG1 expression shifting toward the control profile. *N* = 5 biological replicates per group. (**D**) Ranking of the 14 proteins by adjusted *p*-value and fold change, identifying *Serping1* as the top candidate (**left**) with corresponding relative protein expression levels for *Serping1* across groups (**right**). *N* = 5 mice per group (biological replicates); each symbol represents one mouse. (**E**) Uniform manifold approximation and projection (UMAP) plot of non-myocyte populations from scRNA-seq data, with cell types annotated based on canonical marker genes (**left**) and feature plots showing expression of selected cell-type-specific markers (**right**). Red indicates expression of the indicated marker genes, while grey indicates low or no detectable expression. (**F**) UMAP feature plot showing *Serping1* expression across non-myocyte populations, highlighting enrichment in cardiac fibroblasts (CFs). Panels (**E**,**F**) include pooled scRNA-seq data generated from three experimental groups, with two biological replicates per group, each of which consists of pooled hearts from three mice. (**G**) In vitro validation of *Serping1* enrichment in CFs versus cardiomyocytes (CMs) isolated from neonatal rat hearts, assessed by mRNA (**left**) and protein (middle) expression, with representative Western blot images shown (**right**). *N* = 3 independent biological replicates. Data in panel G represent mean ± SD; statistical significance was assessed using an unpaired two-tailed Student’s *t*-test, with *p* < 0.05 considered significant. Details of proteomics differential analysis and scRNA-seq processing (Cell Ranger/Seurat) are provided in the Methods section.

To investigate the molecular mechanisms underlying the early cardioprotective effects of NRG1 following DOX treatment, we conducted both comprehensive proteomic analysis and single-cell RNA sequencing (scRNA-seq) on heart tissues obtained from Saline (Ctrl), DOX and DOX + NRG1 mice three days post-DOX administration (12 mg/kg). Proteomic profiling provides an exploratory approach to detecting changes in protein expression that may underlie pathophysiological and cardioprotective processes. Across all samples, 5154 proteins were detected. We first compared the proteomic profiles of the Ctrl and DOX-treated groups and identified 184 significantly upregulated and 218 downregulated proteins following DOX treatment (Figure 2A, Supplementary Table S2). To assess the impact of NRG1, we compared the DOX and DOX + NRG1 groups, identifying 58 proteins that were upregulated and 44 that were downregulated in response to NRG1 co-treatment (Figure 2A, Supplementary Table S2). The intersection of the two comparisons identified 23 proteins that were commonly altered by DOX treatment and subsequently modulated by NRG1 (Figure 2B). Of these, 14 proteins that exhibited opposite expression changes between DOX and DOX + NRG1, with expression in DOX + NRG1 shifting toward the control profile, are visualized in Figure 2C. These overlapping proteins are likely key effectors of DOX-induced cardiotoxicity that are responsive to NRG1 intervention. Among these proteins, Serping1 (C1 inhibitor) emerged as the top candidate based on the adjusted *p*-value and fold change (Figure 2D). Serping1 was significantly upregulated by DOX treatment and downregulated by NRG1 co-treatment, suggesting a potential role in mediating early DOX-induced stress responses and their attenuation by NRG1 (Figure 2D).

To determine which cardiac cell types express *Serping1*, we analyzed our scRNA-seq dataset. Using the 10× Genomics platform, we successfully captured all major non-myocyte populations based on established marker genes (Figure 2E). Due to the large size of cardiomyocytes (CMs), which exceeds the capture capability of the 10× Genomics platform, most CMs were intentionally excluded during tissue processing; however, a small number still remained in the dataset. Analysis of *Serping1* expression using scRNA-seq revealed that it is predominantly enriched in cardiac fibroblasts (CFs) (Figure 2F). Next, we used primary CFs and CMs from neonatal rat hearts to evaluate Serping1 expression at the mRNA and protein levels. Rat CFs showed significantly higher Serping1 expression compared with CMs (Figure 2G).

To assess whether *Serping1* expression is also enriched in isolated adult cardiac fibroblasts, we reanalyzed an independent publicly available bulk RNA-seq dataset of sorted adult mouse cardiac cell populations from Quaife-Ryan et al. [26]. This analysis showed that *Serping1* expression was strongly enriched in isolated adult cardiac fibroblasts compared with isolated adult cardiomyocytes, endothelial cells and leukocytes (Supplementary Figure S2), supporting the relevance of Serping1 as a fibroblast-associated gene beyond the neonatal rat culture system.

In summary, our proteomic analysis identifies Serping1 as an early responder to DOX-induced cardiac stress and a target of NRG1-mediated modulation. Its strong enrichment in cardiac fibroblasts suggests a fibroblast-specific role in the early remodeling process triggered by DOX and attenuated by NRG1.

### 2.3. NRG1 Downregulates Serping1, Which Modulates Igf1/Igfbp5 Survival Pathways in Cardiac Fibroblasts

**Figure 3:**
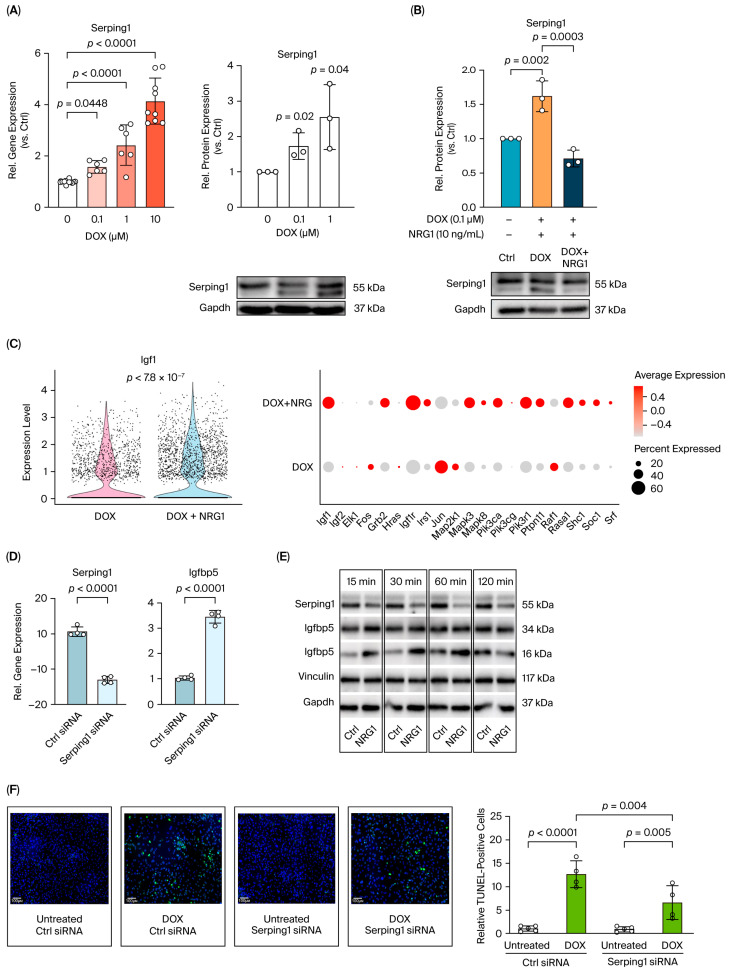
NRG1 attenuates DOX-induced *Serping1* upregulation, leading to modulation of *Igf1*/*Igfbp5* survival pathways in cardiac fibroblasts. (**A**) Dose-dependent induction of *Serping1* mRNA and protein in primary cardiac fibroblasts following treatment with different concentrations of DOX (0.1, 1, or 10 μM) for 24 h. Data are from qPCR (mRNA) and Western blot analysis (protein), with representative blots shown. (**B**) Effect of NRG1 on DOX-induced *Serping1* expression. CFs were treated with DOX (0.1 μM) alone or pre-treated with NRG1 (10 ng/mL, 30 min) prior to DOX exposure for 24 h. *Serping1* protein levels were measured by Western blot; representative blots and quantification are shown. *N* = 3 independent biological replicates (**C**) Violin plot from scRNA-seq analysis of cardiac fibroblasts showing *Igf1* expression levels in DOX vs. DOX + NRG1 groups and expression patterns of Igf pathway components (dot plot, **right**). (**D**) Effect of *Serping1* silencing on *Igfbp5* expression. CFs were transfected with control siRNA or *Serping1* siRNA, and transcript levels of *Serping1* and *Igfbp5* were measured by qPCR. *N* = 3 independent biological replicates. (**E**) Time-course analysis of *Serping1* and *Igfbp5* protein levels in CFs following NRG1 treatment (10 ng/mL). Western blots show *Serping1* and *Igfbp5* in intact (34 kDa) and cleaved (16 kDa) forms at 15, 30, 60 and 120 min. (**F**) TUNEL assay assessing apoptosis in CFs transfected with control siRNA or *Serping1* siRNA under untreated or DOX-treated (0.1 μM, 24 h) conditions. Representative fluorescence images (green: TUNEL-positive; blue: DAPI) and quantification of TUNEL-positive cells are shown, with *N* = 4 independent biological replicates. Scale bar = 100 µm. Data represent mean ± SD; statistical significance was assessed using Student’s *t*-test, one-way ANOVA or two-way ANOVA as appropriate, with *p* < 0.05 considered significant. Bioinformatics and statistical methods for scRNA-seq analysis are described in the Methods section. For *Serping1* Western blot analysis in panels (**A**,**B**), the lower band was used for quantification.

To examine the regulation of Serping1 by DOX and its modulation by NRG1, we performed in vitro assays using primary neonatal rat cardiac fibroblast-enriched cultures. Cells were isolated from neonatal rat hearts by enzymatic digestion, followed by time-controlled differential adhesion/preplating. Specifically, isolated cardiac cells were subjected to two rounds of preplating, each for 45 min, to enrich the rapidly adherent fibroblast fraction and deplete cardiomyocytes from the culture. Experiments were performed using early-passage P1 cells within 24–48 h after isolation to minimize culture-induced changes associated with prolonged expansion. Treatment duration for each experiment is indicated in the corresponding figure legends. To examine the regulation of *Serping1* by DOX and its modulation by NRG1, these cultures were exposed to different concentrations of DOX for 24 h. Our qPCR analysis demonstrated a significant induction of *Serping1* mRNA at 0.1 µM DOX, with further increases observed at 1 µM and 10 µM (Figure 3A). Western blot analysis results consistently confirmed dose-dependent increases in Serping1 protein expression (Figure 3A). To evaluate whether NRG1 attenuates DOX-induced Serping1 upregulation, CF cultures were treated with DOX or DOX + NRG1 and compared with untreated controls. Western blot analysis revealed that DOX significantly increased Serping1 protein levels, whereas NRG1 co-treatment markedly reduced its expression toward control levels (Figure 3B).

To investigate the functional role of Serping1 in CFs, we hypothesized that it may modulate Igf1 signaling by regulating Igfbp5 (Supplementary Figure S3). This rationale was based on a previous report showing that the complement protease C1s cleaves Igfbp5 in human dermal fibroblasts and that Serping1 is a physiological inhibitor of C1s. Thus, it influences the Igf1 pathway activity in dermal fibroblasts via Igfbp5 regulation [27]. To evaluate this potential mechanism, we first analyzed our scRNA-seq dataset and found that *Igf1* expression was significantly higher in cardiac fibroblasts from DOX + NRG1-treated hearts compared with those from the DOX group (Figure 3C, left). Broader examination revealed a trend toward increased expression of multiple Igf pathway genes in the DOX + NRG1 group compared with DOX alone in CFs (e.g., *Igf1*, *Igf1r*, *Irs1*, *Pik3ca* and *Mapk3*) (Figure 3C, right).

To assess the impact of *Serping1* on *Igfbp5* expression in CFs, we silenced *Serping1* using siRNA transfection. As expected, *Serping1* transcript levels were significantly reduced compared with the control siRNA. Interestingly, *Serping1* knockdown resulted in a significant increase in *Igfbp5* expression, indicating that *Serping1* negatively regulates *Igfbp5* in CFs (Figure 3D). Given that NRG1 reduced *Serping1* expression in CFs, we next examined whether Igfbp5 protein levels were also affected following NRG1 treatment. Western blot analysis showed that Serping1 protein levels were significantly reduced at all measured time points (15, 30, 60 and 120 min) compared with the time-matched control condition. Igfbp5 was detected in both its intact form (34 kDa) and as a cleaved fragment (16 kDa). Notably, the NRG1-induced reduction in Serping1 was paralleled by a rapid increase in cleaved Igfbp5 up to 60 min, indicating that Serping1 downregulation facilitates Igfbp5 cleavage in cardiac fibroblasts (Figure 3E).

Since Igfbp5 has been implicated in regulating cell survival and apoptosis [28,29], we next evaluated whether *Serping1* knockdown influences apoptosis in CFs. Apoptotic cell death was assessed using TUNEL staining under control conditions and following DOX exposure in cells transfected with either control siRNA or *Serping1* siRNA. DOX treatment in the control siRNA group significantly increased the proportion of TUNEL-positive CFs compared with untreated controls. Importantly, DOX treatment in the presence of *Serping1* siRNA significantly reduced the proportion of TUNEL-positive CFs compared with DOX plus control siRNA (Figure 3F). These findings support a role for Serping1 in modulating Igf1/Igfbp5-linked survival signaling in cardiac fibroblasts (Supplementary Figure S3).

### 2.4. Expression of SERPING1 in Cardiac Fibroblasts Modulates Cardiomyocyte Contraction Kinetics in Human Cardiac Microtissues

**Figure 4:**
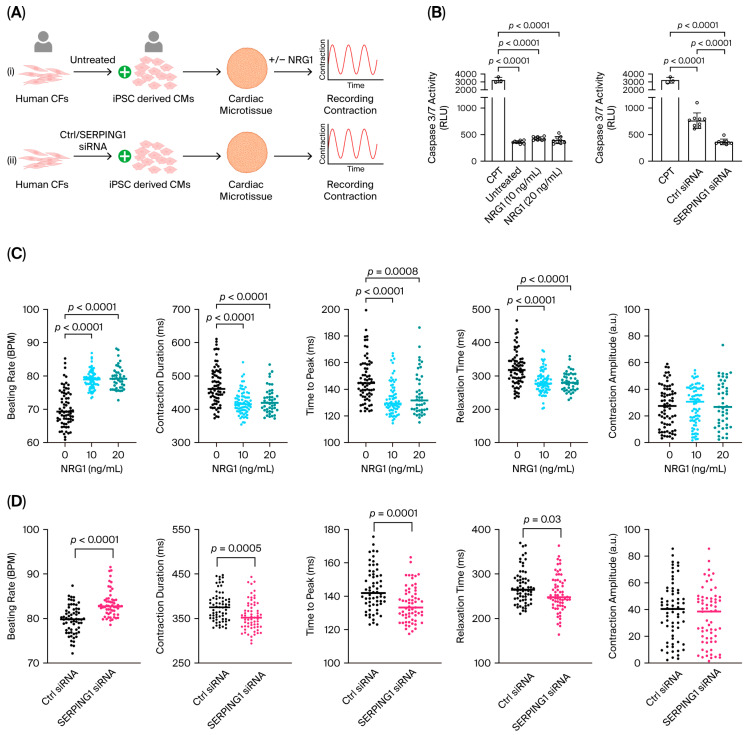
Expression of *SERPING1* in cardiac fibroblasts modulates cardiomyocyte contraction kinetics in human cardiac microtissues. (**A**) Schematic representation of the human cardiac microtissue platform. Scaffold-free, self-aggregating 3D constructs were generated by combining human iPSC-derived cardiomyocytes (hiPSC-CMs) with human cardiac fibroblasts (hCFs) at a fixed 4:1 ratio and cultured for 3 days to enable functional integration (Video S1, Supplemental Data). Fully formed microtissues were either treated with increasing concentrations of NRG1 (**top**) or assembled with hCFs transfected with control or *SERPING1*-targeting siRNA prior to co-culture with hiPSC-CMs (**bottom**). Contractile performance was quantified using MUSCLEMOTION software. (**B**) Caspase 3/7 activity in microtissues following NRG1 treatment (**left**) or in microtissues generated with control vs. *SERPING1* siRNA-transfected hCFs (**right**). (**C**) Effects of NRG1 treatment on microtissue contractile parameters, including beating rate, contraction duration, time to peak, relaxation time and contraction amplitude, with *N* = 183–190 microtissues per group. (**D**) Effects of *SERPING1* knockdown in hCFs on microtissue contractile parameters, with *N* = 120–130 microtissues per group. Data are mean ± SD. Normality was assessed for each dataset; if normally distributed, comparisons were made using ordinary one-way ANOVA; otherwise, the Kruskal–Wallis test was applied. Exact *p* values are shown.

We demonstrated an autocrine role of *Serping1* within CFs, where its expression levels influenced cell survival. We next sought to determine whether *Serping1* also exerts paracrine effects on other cardiac cell types, particularly cardiomyocytes. *SERPING1* encodes a secreted protein released via the endoplasmic reticulum (ER)–Golgi pathway [1,4], but its potential paracrine effects in the heart have not yet been investigated. To address this, we used a human cardiac microtissue platform in which scaffold-free, self-aggregating 3D constructs were generated by combining human iPSC-derived cardiomyocytes (hiPSC-CMs) with human cardiac fibroblasts (hCFs) at a fixed 4:1 ratio and cultured for 3 days to allow functional integration (Supplementary Video File S1) (Figure 4A) [30,31]. Contractile performance was quantified using the MUSCLEMOTION software (manual_v1.0), which enables high-throughput, exploratory analysis of contraction kinetics within microtissues [31].

Interestingly, treatment of fully formed microtissues with NRG1 at either 10 or 20 ng/mL increased beating rates, accompanied by shortened contraction duration, time to peak and relaxation time, without affecting contraction amplitude (Figure 4C). These findings indicate that NRG1 accelerates the speed and frequency of cardiomyocyte contractions in microtissues. Caspase 3/7 activity remained low in both untreated and NRG1-treated microtissues, indicating that the enhanced contractile performance did not lead to cardiomyocyte apoptosis (Figure 4B). These findings are consistent with those of a prior study demonstrating that NRG1 enhances cardiomyocyte contractility in mature cardiomyocytes [32].

To investigate the specific role of fibroblast-derived *SERPING1*, we generated microtissues using hCFs transfected with either control siRNA or *SERPING1*-targeting siRNA before co-culture with hiPSC-CMs (Supplementary Figure S4, showing efficient and stable *SERPING1* knockdown). *SERPING1* knockdown significantly increased beating rate and reduced contraction duration, time to peak and relaxation time, again without altering contraction amplitude (Figure 4D). Importantly, these effects closely mirrored those observed following NRG1 treatment, suggesting that NRG1 may enhance contraction rates, at least in part, by reducing fibroblast-derived *SERPING1*.

Caspase 3/7 activity measurements revealed a modest increase in apoptosis in microtissues assembled with siRNA-transfected hCFs, yet this was attenuated when *SERPING* was silenced, further supporting a possible pro-survival role for reduced *SERPING1* expression (Figure 4B).

Together, these findings suggest that fibroblast-derived *SERPING1* influences cardiomyocyte contractile dynamics in a 3D human microtissue model and demonstrate that either NRG1 treatment or *SERPING1* knockdown in hCFs can enhance contraction kinetics without promoting apoptosis.

### 2.5. NRG1 Treatment Reduces a Wnt-Associated, Erbb4+ Fibroblast Subpopulation Linked to Early Remodeling-Associated Transcriptional Responses

**Figure 5:**
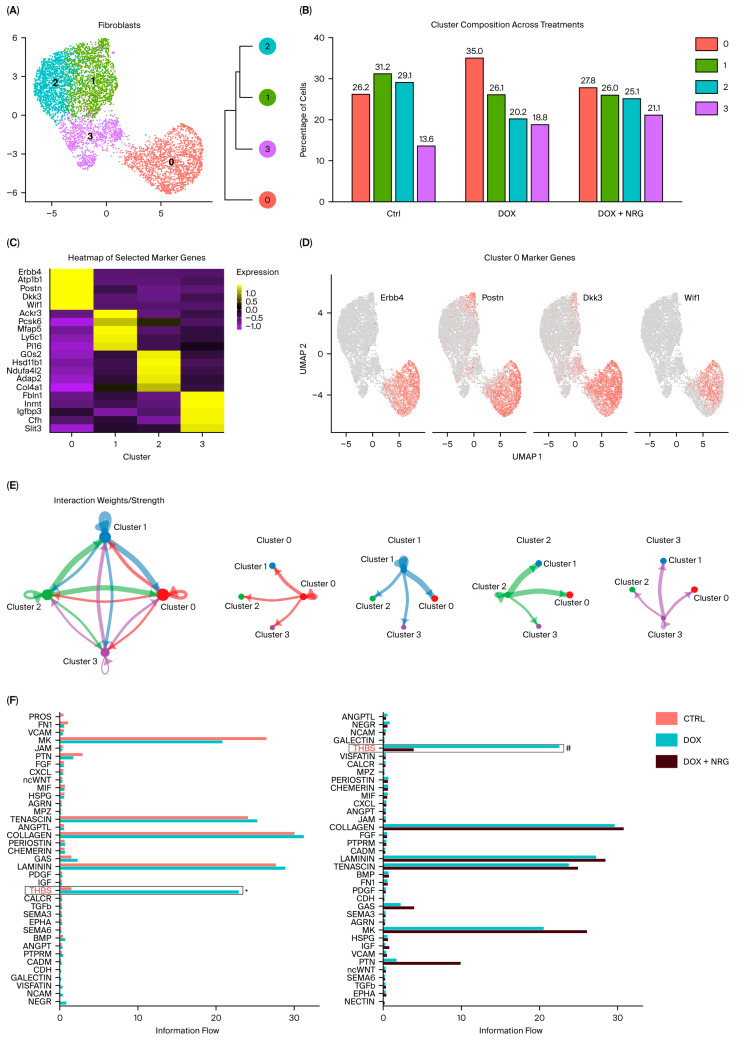
NRG1 treatment reduces a Wnt-associated *Erbb4+* fibroblast subpopulation linked to early pro-fibrotic activation (**A**) UMAP visualization of reclustered cardiac fibroblasts (CFs) from scRNA-seq data, identifying four transcriptionally distinct subclusters (Clusters 0–3). (**B**) Relative abundance of fibroblast subclusters across treatment groups (Ctrl, DOX and DOX + NRG1). (**C**) Heatmap of representative marker genes for each fibroblast subcluster. (**D**) Feature plots illustrating Cluster 0-specific expression of *Erbb4*, *Postn*, *Dkk3* and *Wif1*. Red indicates expression of the indicated marker genes, while grey indicates low or no detectable expression. (**E**) Cell–cell communication analysis with CellChat. Circle plot summarizing aggregate communication among CF subclusters; edge width indicates total (aggregate) communication probability, showing prominent signaling from Clusters 1 and 2 toward Cluster 0. (**F**) Signaling pathway–level differential analysis highlighting THBS (thrombospondin) signaling as the most affected pathway, with increased signaling in DOX vs. Ctrl and attenuation in DOX + NRG1 vs. DOX. In panel (**F**), * and # indicate *p* < 0.001.

To assess whether NRG1 influences early fibroblast activation and fibrosis in DOX-induced cardiotoxicity, we first examined collagen deposition using Picro-Sirius Red staining of paraffin-embedded mouse heart sections (samples from Figure 1). No clear interstitial fibrosis was detected at this early time point (Supplementary Figure S5). To further explore fibrotic signaling, we next investigated fibrosis-related changes in our scRNA-seq dataset. To achieve this, we computationally isolated CFs by excluding all other cell types, thereby enabling a fibroblast-specific analysis and subsequent reclustering. This analysis identified four transcriptionally distinct fibroblast subpopulations (Clusters 0, 1, 2, and 3; Figure 5A). The relative abundance of Cluster 0 fibroblasts differed markedly between treatment groups. It was significantly increased in DOX-treated hearts compared with controls but nearly restored to control levels by NRG1 co-treatment (Figure 5B). Clusters 1 and 3 were unaffected by NRG1, whereas Cluster 2 NRG1 showed a partial recovery from the DOX-induced reduction.

Cluster 0 fibroblasts displayed a transcriptional profile characterized by high levels of expression of secreted Wnt signaling inhibitors (*Wif1* and *Dkk3*), together with *Postn*, a canonical marker of activated CFs (Figure 5C,D). This signature matches the “F-Wnt” fibroblast population recently described in human and mouse hearts [21,22,33], which is thought to represent an intermediate state between fibroblast-transitory (F-Trans) and fully activated fibroblasts (F-Acts). Prior studies show that these Wnt-associated fibroblasts are enriched in injured hearts (e.g., myocardial infarction) and communicate extensively with endothelial cells [22,33].

Importantly, in our dataset, Cluster 0 was the only fibroblast population expressing *Erbb4*, the canonical receptor for NRG1 (Figure 5D and Figure S6). ErbB2 and ErbB3 expression was very low in all fibroblast populations (Supplementary Figure S6). Thus, NRG1 may directly signal Cluster 0 cells via ErbB4.

Taken together, although histological analysis revealed no clear fibrosis at this early DOX time point, our single-cell transcriptomic data identify a distinct, partially activated fibroblast subpopulation defined by *Postn* and secreted Wnt inhibitors that expands early after DOX exposure and is selectively reduced by NRG1 treatment. These findings suggest that NRG1 protects the heart, partly by preventing the early differentiation of fibroblasts toward a pro-fibrotic intermediate state.

We further profiled communication among CF subclusters using CellChat. CellChat estimates communication probability from ligand levels in senders and receptor/co-receptor levels in receivers. Signals are then aggregated across cells within each cluster [34]. CellChat analysis showed prominent outgoing signals from Clusters 1 and 2 toward Cluster 0, with thicker lines indicating higher aggregate communication probability (Figure 5E). This indicates that Cluster 0 functions as a major signal receiver within the CF network. We then examined which signaling pathways drive these inputs across groups. Signaling pathway-level differential analysis identified THBS (thrombospondin) signaling as the most affected, showing an increase in DOX vs. Ctrl and attenuation in DOX + NRG1 vs. DOX (Figure 5F). Given the established pro-fibrotic roles of thrombospondin signaling in cardiac remodeling [35,36,37,38], these findings provide supportive evidence that NRG1 mediates an early anti-fibrotic effect.

### 2.6. NRG1 Has Limited Impact on Systemic Inflammatory Responses to DOX

We next examined whether DOX induced systemic inflammation in our mouse model and whether NRG1 modified this response. We used the Meso Scale Discovery (MSD; K15713K; Meso Scale Discovery, Rockville, MD, USA) U-PLEX^®^ Proinflammatory Combo 1 (Mouse) panel, which enables precise, absolute quantification of 10 key proinflammatory cytokines and chemokines in serum. The analytes included *IFN-γ*, *IL-1β*, *IL-2*, *IL-6*, *IL-10*, *IL-12p70*, *IL-17A*, *IP-10*, *MCP-1* and *TNF-α*. Serum samples were collected from the same experimental groups described in Figure 1.

Among the 10 analytes, five showed significant differences between the groups, while the remaining cytokines were either below the detection limit or unchanged (Supplementary Figure S7). DOX treatment significantly increased *MCP-1*, *TNF-α*, *IFN-γ*, *IL-12p70* and *IL-2* compared with saline controls. NRG1 co-treatment did not significantly alter *TNF-α*, *IFN-γ*, *IL-12p70* or *IL-2* levels relative to DOX alone. Notably, *MCP-1* was the only inflammatory mediator significantly reduced by NRG1 in the DOX-treated mice (Supplementary Figure S6). This finding indicates that while NRG1 does not broadly suppress early systemic cytokine responses to DOX, it may attenuate specific chemokine-driven inflammatory processes.

## 3. Discussion

This study demonstrates that NRG1 maintains cardiac function during DOX treatment without compromising its anti-tumor efficacy, a crucial finding for its therapeutic application in cancer patients. By integrating untargeted left-ventricular proteomics with single-cell transcriptomics, we identify *Serping1* as a novel fibroblast-enriched mediator of early DOX stress that is downregulated by NRG1. Mechanistic interrogation supports a model in which Serping1 regulates Igf1/Igfbp5 signaling in cardiac fibroblasts, with downstream effects on fibroblast survival and paracrine modulation of cardiomyocyte contraction kinetics. Concomitantly, NRG1 selectively reduces an *Erbb4+* Wnt-associated fibroblast subpopulation linked to an early pro-fibrotic transcriptional program. Together, these findings extend the concept of NRG1 cardioprotection beyond direct cardiomyocyte signaling to include important fibroblast-mediated mechanisms that influence cardiac responses to anthracycline injury.

Our findings are consistent with previous reports showing that NRG1 protects against doxorubicin-induced cardiotoxicity [23,24,25]. However, most previous studies focused primarily on cardiomyocyte-centered mechanisms. Our study extends this literature by identifying a fibroblast-centered, Serping1-associated response and by integrating in vivo proteomics, single-cell RNA-seq, primary cardiac fibroblast-enriched cultures and human cardiac microtissues. In addition, the use of a tumor-bearing breast cancer model allowed us to evaluate cardioprotection while simultaneously assessing whether NRG1 interferes with doxorubicin anti-tumor efficacy.

Our in vivo data demonstrate that DOX exposure results in systolic dysfunction and pathological remodeling, including chamber dilation and reduced relative wall thickness, which is consistent with clinical reports of anthracycline cardiotoxicity [7,39,40]. Importantly, daily administration of NRG1 preserved the ejection fraction and attenuated structural remodeling while maintaining DOX’s antitumor efficacy. This finding is in line with prior evidence that recombinant human NRG1 improves left ventricular function in patients with heart failure [15,16]. Notably, whereas most preclinical cardio-oncology studies have investigated cardioprotective drugs in healthy mice [23,41,42], we deliberately employed a tumor-bearing breast cancer model to reflect a clinically relevant scenario in which any cardioprotective intervention must preserve the antitumor activity of chemotherapy. This issue is particularly relevant for NRG1 because it activates ErbB receptor signaling, which may potentially support growth or survival pathways in tumors that depend on ErbB signaling.

Mechanistically, our proteomic analysis revealed Serping1 (complement C1 inhibitor; serine proteinase inhibitor) as one of the most strongly induced proteins following DOX exposure, with subsequent downregulation by NRG1 co-treatment. Single-cell transcriptomic analysis of non-cardiomyocyte populations further localized *Serping1* expression to cardiac fibroblasts. In vitro experiments confirmed its enrichment in fibroblasts relative to cardiomyocytes. This is a notable finding, as Serping1 has not previously been implicated in anthracycline cardiotoxicity or in NRG1-mediated cardioprotection. There is limited information regarding Serping1 in heart tissue. Serping1 was recently reported as a novel biomarker associated with poor coronary collateral circulation in patients with stable coronary disease and chronic total occlusion [43]. Experimental work in rats suggests that a C1 inhibitor can be produced locally in the heart following acute myocardial infarction [44]. Serping1 serves as a physiological inhibitor of C1, thereby controlling classical complement activation [45]. Beyond immune regulation, Serping1 can indirectly influence growth factor pathways, as C1 cleaves Igfbp5, a regulator of Igf1 bioavailability [27]. By suppressing Serping1, NRG1 may facilitate Igfbp5 cleavage and thereby enhance Igf1 pathway activity, promoting fibroblast survival and stress adaptation. Future studies assessing IGF1 receptor signaling, including Akt/ERK activation and IGF1R inhibition or rescue experiments, will be required to determine whether the Igf1/Igfbp5 axis directly mediates the fibroblast survival phenotype associated with Serping1 modulation.

Furthermore, our microtissue experiments revealed that Serping1 exerts paracrine effects on cardiomyocytes, modulating contractile kinetics. Human cardiac microtissues assembled from hiPSC-derived cardiomyocytes and fibroblasts showed accelerated contraction dynamics following NRG1 treatment or *Serping1* knockdown in fibroblasts without increases in apoptosis. This indicates that fibroblast-derived Serping1 regulates cardiomyocyte performance, expanding the traditional view of fibroblasts as passive contributors to extracellular matrix turnover. Indeed, accumulating evidence suggests that fibroblasts actively influence cardiomyocyte electrophysiology and contractility via paracrine and juxtacrine signaling [17,46,47,48]. By linking fibroblast-derived Serping1 to contractile modulation, our data reinforce the concept that fibroblast–cardiomyocyte communication is an important determinant of cardiac performance. Clinically, this highlights fibroblasts as actionable therapeutic targets in preventing chemotherapy-induced cardiotoxicity, a concept supported by recent single-cell atlases that reveal fibroblast heterogeneity and dynamic reprogramming during cardiac injury [20,21,33,49].

Consistent with this view, our single-cell RNA-seq analysis identified a distinct *Erbb4+* fibroblast subpopulation characterized by Wnt signaling inhibitors and *Postn* expression, which expanded in response to DOX but was reduced by NRG1. This population resembles the recently described F-Wnt fibroblasts, an intermediate state between quiescent and fully activated fibroblasts that emerges early after cardiac injury [21,22,33]. The selective expression of *Erbb4* in this subcluster suggests that NRG1 may act directly on this subpopulation to prevent its transition toward a pro-fibrotic phenotype. Although we did not detect histological fibrosis at this early time point, the scRNA-seq data provide a molecular signature of early fibroblast activation that could, if unchecked, contribute to late fibrotic remodeling. These findings indicate that NRG1 acts not only as an acute cardioprotectant but also as a modulator of long-term fibroblast fate decisions. Given the central role of fibrosis in anthracycline cardiomyopathy [50,51,52], interventions that block the fibroblast transition toward a pro-fibrotic state may provide lasting benefit.

A limitation of our study is that we mainly examined the early phase of DOX injury; therefore, the longer-term effects of NRG1 in the context of DOX on cardiac structure and function were not addressed. Moreover, because NRG1 treatment was initiated concurrently with DOX exposure, the present study primarily models preventive/early cardioprotection rather than therapeutic reversal after established cardiotoxicity. Therefore, whether NRG1 can reverse established DOX-induced cardiac dysfunction or modify delayed/chronic anthracycline-induced remodeling remains to be determined in future studies. The 4T1 tumor model provides a clinically relevant platform, but it cannot capture the full diversity of human cancers or treatment regimens. In addition, our findings are most applicable to ErbB-negative tumors and may not extend to ErbB-positive cancers. Another limitation is that part of the mechanistic in vitro work was performed using primary neonatal rat cardiac fibroblast-enriched cultures. Neonatal rat cardiac fibroblasts are a well-established primary cell model for controlled mechanistic studies; however, they do not fully recapitulate adult cardiac fibroblast biology in vivo. In addition, direct marker-based validation of fibroblast purity was not performed for every preparation in the present study; therefore, we refer to these cultures as primary neonatal rat cardiac fibroblast-enriched cultures. Future work should therefore extend our findings to long-term preclinical studies, incorporate patient-derived samples and validate the functional role of *Serping1* in freshly isolated adult mouse and human primary cardiac fibroblasts.

## 4. Materials and Methods

### 4.1. Animal Experiments

All animal procedures were conducted under a valid license from the Veterinary Office of the Canton of Basel, Switzerland, in strict accordance with the Swiss Federal Act on Animal Protection. All protocols were approved by the local authorities (License No. 2548_34188 for mice; License No. 2544_36289 for rats) and complied with Directive 2010/63/EU of the European Parliament on the protection of animals used for scientific purposes.

### 4.2. Treatment of Tumor-Bearing Mice with DOX and NRG1

Before in vivo implantation, 4T1 breast tumor cells were maintained in the exponential growth phase. A dose of 0.1 mg/kg of buprenorphine (Bupaq^®^, VetViva Richter GmbH, Wels, Austria) was administered subcutaneously to 8–11-week-old female BALB/c mice (Janvier Lab; Le Genest-Saint-Isle, France) one hour before tumor cell implantation, and subsequently every 4–6 h during the daytime. Additional buprenorphine was provided in the drinking water (9.375 mg/L) during the night for 48 h after implantation. For tumor cell injection, mice were anesthetized with 3% isoflurane carried in 95% oxygen. An ophthalmic lubricant (Lacrinorm^®^, Bausch & Lomb Swiss AG, Zug, Switzerland) was applied to the eyes to prevent corneal drying during anesthesia. The surgical area was shaved to remove hair and disinfected with povidone-iodine (Betadine^®^, Mundi Pharma, Basel, Switzerland), followed by application of a wound disinfectant spray (Octenisept^®^, Schülke & Mayr GmbH, Frauenfeld, Switzerland). A small incision (5–10 mm) was made to gently expose the mammary gland. The cell suspension (1 × 10^5^ tumor cells in 50% Matrigel) was then slowly injected into the gland. Successful injection was confirmed when a small bubble appeared in the gland’s fatty tissue. The gland was repositioned by pulling the skin around the incision, and the incision was closed using 1–2 clips (1.75 × 7.5 mm). Sulfamethoxazole/trimethoprim (160/32 mg/L, Bactrim^®^, Eumedica Pharmaceuticals AG, Basel, Switzerland) was provided in the drinking water for 48 h after tumor cell implantation to prevent wound infection. One week after cell injection, DOX (Doxorubicin hydrochloride, Pfizer, Zürich, Switzerland) was administered intraperitoneally at 3 mg/kg on days 0, 3, 6, and 9. NRG1 (20 µg/kg, Peprotech, Hamburg, Germany) or saline was also administered intraperitoneally, starting one day before the first DOX injection and continued daily until the end of the experiment. The NRG1 dose of 20 µg/kg was selected based on previous preclinical studies demonstrating cardioprotective effects of recombinant NRG1 at this dose range in doxorubicin-induced cardiotoxicity and other cardiac remodeling models [25,53]. Body weight and tumor size were monitored over time as primary outcomes. Tumor volumes were calculated using the formula V = (W^2^ × L)/2, where V is volume, W is width, and L is length, based on caliper measurements. Echocardiography was performed at baseline (before tumor cell injection) and before sacrifice. At the end of the experiment, mice were placed under deep anesthesia using 5% isoflurane in 95% oxygen. Upon confirmation of surgical plane anesthesia, the animals were euthanized by rapid excision of the heart. The hearts were collected for molecular and cellular analyses.

### 4.3. Echocardiography

Transthoracic echocardiography was conducted using a Vevo 2100 Ultrasound system (VisualSonics, Toronto, ON, Canada) equipped with an MS-550 linear-array probe working at a central frequency of 40 MHz. Measurements were performed at baseline (before tumor cell injection) and before sacrifice. Anesthesia was induced with 3% isoflurane/95% oxygen in an induction chamber and maintained with 1.5% isoflurane through a nose cone. An eye ointment (Lacrinorm^®^, Vaughan, ON, Canada) was applied to prevent corneal drying during anesthesia. Body temperature was monitored by a rectal thermometer and maintained at around 37 °C. Chest hair was removed with a depilatory cream (Nair^TM^, Church and Dwight Co., Toronto, ON, Canada). Parasternal long-axis views in B-mode and short-axis views at mid-papillary muscle level in both B- and M-mode were acquired. Data were analyzed with Vevo 2100 1.6.0 software. The LV anterior wall (LVAW), posterior wall (LVPW) thickness and internal diameter (LVID) were derived from the M-Mode measurements for end systole (s) and diastole (d). Values are averages of three consecutive cardiac cycles. LV volume (Vol) was computed based on the Teichholz formulas, and LV ejection fraction (EF) was calculated from the derived LV Vol. LV Vol;d = (7.0/(2.4 + LVIDd)) × LVIDd [54]; LV Vol;s = (7.0/(2.4 + LVIDs)) × LVIDs [54]; EF = 100 × ((LV Vol;d − LV Vol;s)/LV Vol;d). LV mass was calculated based on the corrected cube formula: LV mass = 1.053 × [(LVIDd + LVPWd + LVAWd)3 − LVIDd3] × 0.8. Relative wall thickness (RWT) was calculated for both systole and diastole as RWT = (2 × LVPW)/LVID, expressed as RWTs and RWTd, respectively.

### 4.4. Proteomic Sample Preparation and Analysis

A single dose of DOX (12 mg/kg, i.p.) was injected into female BALB/c mice (8–11 weeks old, non-tumor bearing). NRG1 (20 μg/kg, i.p.) was administered once daily, starting one day before DOX injection and continued until the end of the experiment. Three days after DOX treatment, mice were euthanized as described above, and the hearts were collected. A comprehensive proteomics analysis was conducted on the left ventricle (LV) from Ctrl, DOX, and DOX plus NRG1 groups (*N* = 5/group).

LV samples were lysed in 5% SDS and 100 mM Triethylammonium Bicarbonate using mechanical disruption, followed by 10 min of sonication with Pixul™ (LubioScience GmbH, Zürich, Switzerland) (Pulse 50 N, PRF 1 kHz, burst rate 20 Hz). After tissue disruption, TCEP was added to a final concentration of 10 mM, and samples were incubated for 10 min at 95 °C. Proteins were alkylated using 20 mM iodoacetamide at 25 °C in the dark for 30 min. Proteins (20 µg per sample) were purified and digested using the SP3 approach [55] with a Freedom Evo 100 liquid handling platform (Tecan Group Ltd., Männedorf, Switzerland).

In brief, Speed Beads™ (#45152105050250 and #65152105050250, GE Healthcare, Opfikon, Switzerland) were mixed 1:1, rinsed with water, and diluted to an 8 µg/µL stock solution. Samples were adjusted to a final volume of 90 µL, and 10 µL of the bead stock solution was added. Proteins were bound to the beads by adding 100 µL of 100% acetonitrile to the samples, which were then incubated for 8 min at room temperature with gentle agitation (200 rpm). Afterward, samples were placed on a magnetic rack and incubated for 5 min. Supernatants were removed and discarded. The beads were washed twice with 160 µL of 70% (*v*/*v*) ethanol and once with 160 µL of 100% acetonitrile.

The samples were then placed off the magnetic rack, and 50 µL of digestion mix (10 ng/µL trypsin in 50 mM triethylammonium bicarbonate) was added. Digestion was allowed to proceed for 12 h at 37 °C. After digestion, samples were placed back on the magnetic rack and incubated for 5 min. Supernatants containing peptides were collected and dried under vacuum.

Peptides were resuspended in 0.1% aqueous formic acid, and 0.2 µg of peptides subjected to LC–MS/MS analysis using an Orbitrap Exploris 480 Mass Spectrometer fitted with a Vanquish Neo (both Thermo Fisher Scientific, Basel, Switzerland) and a custom-made column heater set to 60 °C. Peptides were resolved using a RP-HPLC column (75 μm × 30 cm) packed in-house with C18 resin (ReproSil-Pur C18–AQ, 1.9 μm resin; Dr. Maisch GmbH, Ammerbuch, Germany) at a flow rate of 0.2 μLmin-1. Separation of peptides was achieved using the following gradient: 4% Buffer B to 10% Buffer B in 5 min, 10% Buffer B to 35% Buffer B in 45 min, and 35% Buffer B to 50% Buffer B in 10 min. Buffer A was 0.1% formic acid in water, and buffer B was 80% acetonitrile, 0.1% formic acid in water.

The mass spectrometer was operated in DIA acquisition mode with a total cycle time not exceeding approximately 3 s. For MS1, the following parameters were set: Resolution: 120,000 FWHM (at 200 *m*/*z*), Scan Range: 390–9190 *m*/*z*, Injection time: auto, Normalized AGC Target: 300%. MS2 (SWATH) scans were acquired using the following parameters: Isolation Window: 14 *m*/*z*, HCD Collision Energy (normalized): 28%, Normalized AGC target: 1000%, Resolution: 15,000 FWHM (at 200 *m*/*z*), Precursor Mass Range: 400–100 *m*/*z*, Max. Fill Time: 22 ms, DataType: Centroid.

The acquired raw files were searched using SpectroNaut (v18.3, directDIA workflow, default settings) against a murine database (downloaded from Uniprot on 22 February 2022) using the following search criteria: full tryptic specificity was required (cleavage after lysine or arginine residues, unless followed by proline); 3 missed cleavages were allowed; carbamidomethylation (C) was set as fixed modification; oxidation (M), N-acetylation (N-term). The identified transitions were exported as tsv files and processed using the MSstats R package v4.0.1 [56]. Downstream analyses were performed using Qlucore Omics Explorer (base module, v3.8).

### 4.5. Single-Cell RNA Sequencing and Data Analysis

A single intraperitoneal dose of DOX (12 mg/kg) was administered to female BALB/c mice (8–11 weeks old; non–tumor-bearing; Janvier, Le Genest-Saint-Isle, France). NRG1 (20 µg/kg, i.p.) was given once daily, starting one day before NOX injection and continuing until the end of the experiment. Three days after DOX treatment, mice were placed under deep anesthesia using 5% isoflurane in 95% oxygen. Upon confirmation of surgical plane anesthesia, the animals were euthanized by rapid excision of the heart. To isolate non-myocyte cells for single-cell sequencing, beating adult mouse hearts of 3 experimental groups (two biological replicates per group, each replicate consisting of pooled hearts from three mice) were dissected and placed in ice-cold HBSS without Ca^2+^ and Mg^2+^. The atria and large vessels were discarded, and the ventricles were minced. The tissue was digested using a solution containing Pierce Primary Cardiomyocyte Enzyme (Thermo Scientific, Waltham, MA, USA, cat. 88281) that was perfused through the ventricles, followed by a 10 min incubation at 37 °C. This digestion cycle was repeated 2–3 times. The tissue was then pipetted up and down 15–20 times to generate a single-cell suspension, which was passed through a 300-µm strainer. Cells were gently centrifuged (200 rpm, 5 min) to remove cardiomyocytes. The supernatant was collected and passed through a 40-µm strainer. Non-myocyte cells were pelleted by centrifugation at 800 rpm for 5 min and resuspended in DMEM containing 10% FBS.

Fluorescence-activated cell sorting (FACS) was performed to enrich for metabolically active, viable non-myocyte cells using Calcein AM^+^ and DAPI staining. Sorted cells were processed on the 10× Genomics Chromium platform. Cells were loaded into a Chromium Next GEM Chip (10× Genomics) and sequencing libraries were prepared according to the manufacturer’s protocol. Barcoded gel beads were combined with cells and partitioning oil to generate tens of thousands of single-cell emulsions. Barcoded fragments were pooled for downstream reactions, producing libraries compatible with short-read sequencing. Libraries were quantified and assessed on a Fragment Analyzer (Agilent Technologies, Basel, Switzerland). Sequencing was performed on an Illumina HiSeq 4000 using HiSeq 3000/4000 SBS Kit reagents (Illumina, Inc., San Diego, CA, USA).

Sequencing data were demultiplexed with bcl2fastq2 Conversion Software (v2.20, Illumina). Cell Ranger (v1.3, 10× Genomics) was used to generate FASTQ files from raw scRNA-seq data. Reads were aligned to the mm10 reference genome with STAR (v2.6.0c) using the UCSC mm10 gene annotation (GTF). Downstream bioinformatics analysis was performed in R using Seurat (v3) [40]. Dimensionality reduction was performed with the RunUMAP function in Seurat, implementing the Uniform Manifold Approximation and Projection (UMAP) algorithm. Differentially expressed genes for each cluster were identified using the FindAllMarkers function with the Wilcoxon test, and *p*-values were corrected for multiple testing using the Bonferroni method. Gene expression was visualized with the FeaturePlot, DoHeatmap, and DotPlot functions in Seurat [40].

### 4.6. Cardiomyocyte (CM) and Non-Cardiomyocyte (Non-CM) Isolation from Neonatal Rat Hearts

Primary cultures were prepared from one to two-day-old Sprague Dawley rats. Neonates were euthanized by decapitation, after which the hearts were rapidly excised, and atria and major vessels were removed. Ventricles were incubated overnight at 4 °C in 0.05% trypsin/EDTA (Gibco, cat. 25300-054, Thermofisher, Basel, Switzerland) with gentle agitation. The following day, residual trypsin was discarded, and ventricles were washed once in DMEM containing 10% fetal bovine serum (FBS) (Gibco, cat. 10270-106) for 4 min at 37 °C with gentle agitation. Ventricles were then digested in collagenase type II (0.86 mg/mL; Worthington, cat. LS004177, Lakewood, NJ, USA) in HBSS (Gibco, cat. 14170-088) at 37 °C with gentle agitation [57]. This step was repeated 3–4 times for 2–3 min each until cells were dissociated. Cell suspensions were centrifuged, and the pellets were resuspended in low-glucose DMEM (1 g/L glucose) supplemented with 10% FBS, 1% penicillin/streptomycin (Gibco, cat. 10378-016) and 25 mM HEPES (Gibco, cat. 15630-056).

To enrich the cardiomyocyte fraction, cells underwent two consecutive pre-plating steps (45 min each at 37 °C, 5% CO_2_), during which non-cardiomyocytes adhered rapidly to the flask surface. The supernatant enriched in CMs was collected, counted and plated for experiments. Adherent non-CMs were maintained in complete DMEM, containing high-glucose (4.5 g/L), 10% FBS and 1% penicillin/streptomycin for subsequent experiments.

This time-controlled differential adhesion/pre-plating step was used to enrich the rapidly adherent fibroblast fraction and deplete cardiomyocytes from the adherent non-CM culture. This approach is widely used to enrich neonatal cardiac fibroblasts from mixed cardiac cell preparations after enzymatic dissociation [54,58,59,60], although individual protocols may differ in the specific digestion enzymes, digestion steps, or preplating duration used. The non-CM/fibroblast-enriched fraction was maintained in high-glucose DMEM supplemented with 10% FBS and 1% penicillin/streptomycin. These culture conditions are commonly used for cardiac fibroblast maintenance and are not specifically optimized to support endothelial cell or leukocyte expansion. Cells were passaged once, and all experiments using fibroblast-enriched cultures were performed with early-passage P1 cells within 24–48 h after isolation. Treatment duration and experimental conditions are specified in the corresponding figure legends.

### 4.7. Treatment of Neonatal Rat Non-Cardiomyocytes with DOX and NRG1

After reaching confluency following 24–48 h of incubation post-isolation, non-CMs were incubated in complete high-glucose DMEM as follows. For pre-treatment, cells were washed once with PBS and incubated for 30 min with NRG1 (R&D Systems, cat. 377-HB-050, Minneapolis, MN, USA) diluted in complete DMEM to final concentrations of 10 or 20 ng/mL. Without replacing the medium, doxorubicin (DOX; LC Laboratories, cat. D-400, Woburn, MA, USA) was then added to the NRG1-containing medium to achieve final concentrations ranging from 0.1 to 10 µM. Control wells received complete DMEM without additives; DOX-alone wells received DOX without NRG1; NRG1-alone wells received NRG1 without DOX. Treatment durations ranged from 15 min to 24 h as specified in the figure legends. After treatment, cells were processed for protein analysis, RNA analysis or microscopy.

### 4.8. RNA Isolation, cDNA Synthesis and Quantitative RT-PCR Analysis

Total RNA was isolated from tissues or cultured cells using the RNeasy Plus Universal Mini Kit (Qiagen, cat. 73404, Steinhausen, Switzerland) or RNeasy Micro Kit (Qiagen, cat. 74004), respectively. Complementary (c)DNA was synthesized using the High-Capacity cDNA Reverse Transcription Reagents Kit (Applied Biosystems, cat. 4368814, Waltham; MA, USA). Quantitative RT-PCR (qRT-PCR) was performed using the GoTaq^®^ RT-qPCR Reagents Kit (Promega, cat. A6002, Dübendorf, Switzerland) with gene-specific primers (Supplementary Table S3). Raw values were normalized to Gapdh. Mean fold differences were calculated using the comparative Ct method (2^−∆∆Ct^).

### 4.9. Protein Extraction and Western Blotting

After treatment, cells were washed with ice-cold PBS and lysed in RIPA buffer supplemented with protease and phosphatase inhibitors. Lysates were incubated on ice, centrifuged at 12,700 rpm for 10 min at 4 °C, and supernatants were collected. Protein concentration was determined using the Pierce Micro BCA Protein Assay Kit (Thermo Scientific, Waltham, MA, USA).

Equal amounts of protein were denatured in Laemmli buffer, separated by SDS–PAGE and transferred onto PVDF membranes. Membranes were blocked in 5% milk in TBS-T, incubated overnight at 4 °C with primary antibodies against *Serping1* (Abcam, ab229209, Cambridge, UK), *Igfbp5* (Abcam, ab251234), Gapdh (Santa Cruz, sc-32233, Dallas, TX, USA) or Vinculin (Santa Cruz, sc-73614), and then with HRP-conjugated secondary antibodies for 1 h at room temperature. Proteins were detected using SuperSignal West Pico PLUS chemiluminescent substrate (Thermo Scientific, Waltham, MA, USA) and imaged with a Vilber Fusion FX system. Band intensities were quantified using Fiji software (v1.53k).

### 4.10. TUNEL Assay

Apoptosis was assessed using the In Situ Cell Death Detection Kit, TMR red (Roche, cat. 12156792910, Basel, Switzerland) according to the manufacturer’s protocol. Briefly, cardiac fibroblasts were fixed with 4% formaldehyde, permeabilized and incubated with the TUNEL reaction mixture for 60 min at 37 °C in a humidified dark chamber. After washing, nuclei were counterstained with DAPI (1:1000 in PBS) for 10 min at room temperature. Samples were mounted and imaged under a fluorescence microscope (excitation 450–500 nm; emission 515–565 nm). Quantification of TUNEL-positive (red) versus total nuclei (blue) was performed using QuPath (v0.5.0).

### 4.11. Small Interfering RNA (siRNA)-Mediated Gene Silencing

Rat cardiac fibroblasts were transfected with FlexiTube siRNA targeting rat *Serping1* (SI02021257|S1; Rn_RGD:735225_3, Qiagen, Steinhausen, Switzerland) or a non-targeting scrambled control (siCtrl) using Lipofectamine 3000 (Thermo Fisher Scientific, Basel, Switzerland) according to the manufacturer’s instructions. Transfections were carried out in serum-free Opti-MEM (Invitrogen, Basel, Switzerland) in 12-well plates for 48 h. Cells were then used for functional assays or for quantification of target gene expression following treatment with DOX or DOX + NRG1.

### 4.12. Picro-Sirius Red Staining

For Picro-Sirius Red staining, paraffin-embedded mouse heart tissue sections were first deparaffinized in Ultraclear (3 times, 2 min each), followed by rehydration through a graded ethanol series (100%, 70%, and 60% ethanol, each step 2 times for 2 min). Sections were then rinsed in tap water for 30 s. The sections were stained with Picro-Sirius Red solution (ScyTek Laboratories, SR500, Logan, UT, USA) for 1 h, washed in 1% acetic acid for 2 min to remove excess stain and dehydrated through graded ethanol (96% and 100% ethanol, 5 min each). Finally, the sections were cleared in Ultraclear and mounted using a permanent mounting medium. This protocol was used to visualize collagen fibers in heart tissue under light microscopy.

### 4.13. Human Cardiac Microtissue Assembly and Functional Analysis

Human induced pluripotent stem cell-derived cardiomyocytes (hiPSC-CMs; Cellular Dynamics International, Tokyo, Japan) and human cardiac fibroblasts (hCFs; Sigma, Darmstadt, Germany) were used to generate scaffold-free microtissues. Fibroblasts were cultured in standard growth medium and used at passages 14–17. Where indicated, hCFs were transfected with either non-targeting control or *SERPING1*-specific siRNA using Lipofectamine 2000 prior to microtissue assembly. Microtissues were formed by combining hiPSC-CMs and hCFs at a 4:1 ratio (5000 cells/well) in ultra-low attachment 96-well plates and cultured for 3 days in maintenance medium to allow self-aggregation and functional coupling [30,31]. For NRG1 studies, fully assembled microtissues were treated with recombinant human NRG1 at two concentrations. Contractile performance was recorded in live cultures and analyzed using the MUSCLEMOTION algorithm to extract beating rate, contraction duration, time to peak, relaxation time and amplitude [31]. Apoptotic activity was quantified in parallel samples using the Caspase-Glo 3/7 assay (Promega).

### 4.14. Quantification of Proinflammatory Responses

Plasma concentrations of 10 proinflammatory cytokines and chemokines were measured using the Meso Scale Discovery (MSD) U-PLEX^®^ Proinflammatory Combo 1 (Mouse) panel (K15713K; Meso Scale Discovery, Rockville, MD, USA) following the manufacturer’s instructions. Analytes included *IFN-γ*, *IL-1β*, *IL-2*, *IL-6*, *IL-10*, *IL-12p70*, *IL-17A*, *IP-10* (CXCL10), *MCP-1* (CCL2) and *TNF-α*. Assays were performed on 30 µL of plasma per sample. Plasma samples were collected from the experimental groups described in Figure 1.

### 4.15. Statistical Analysis

Statistical analysis was performed using GraphPad Prism^®^ V10 (GraphPad software, Boston, MA, USA). Data throughout the paper are expressed as mean ± SD. The Shapiro–Wilk test was used to assess the assumption of normality in the datasets. When appropriate, we log-transformed the data to obtain normality in the distributions and performed statistical analysis on log-transformed data. The equality of variances was checked using the Brown-Forsythe test or Spearman’s test for heteroscedasticity in a one-way ANOVA and two-way ANOVA, respectively. Student’s *t*-test, Welch’s *t*-test, one-way ANOVA and two-way ANOVA were used as indicated in the legends.

## Data Availability

All data supporting the findings of this study are included in the article and its Supplementary Information. Additional data, including scRNA-seq data, are available from the corresponding author upon reasonable request.

## Supplementary Materials

The following supporting information can be downloaded at https://www.mdpi.com/article/10.3390/ijms27104616/s1.

## Abbreviations

DOX	doxorubicin
NRG1	Neuregulin-1β
EF	ejection fraction
LVAW	left ventricular anterior wall
LVPW	left ventricular posterior wall
LVID	left ventricular internal diameters
LV Vol	ventricular volumes
RWT	relative wall thickness
i.p.	intraperitoneally
scRNA-seq	single-cell RNA sequencing
*Serping1*	C1 inhibitor
CF	cardiac fibroblast
hiPSC-CMs	human iPSC-derived cardiomyocytes
hCFs	human cardiac fibroblasts
