# Supplementary material for: Neuregulin-1β Mitigates Doxorubicin-Induced Cardiotoxicity via Serping1 in Cardiac Fibroblasts
Source: Int J Mol Sci. 2026 May 21;27(10):4616. doi: 10.3390/ijms27104616 (PMC13207643; doi:10.3390/ijms27104616)
Supplement: Supplementary file 1 [file ijms-27-04616-s001.zip › Supplemental Figures_V2.pdf]

Supplemental Methods, Figures, and Tables

Neuregulin-1β Mitigates Doxorubicin-Induced Cardiotoxicity via *Serping1* in Cardiac Fibroblasts

Parisa Aghagolzadeh [1,*], Lifen Xu [1], Philipp Klinger [1], Christian Morandi [1], Lilia Maryse Lépine [1], Lukas Minder [2],
Pieter-Jan Guns [3], Matthias Bosman [3], Marie-May Coissieux [4], Thierry Pedrazzini [5], Gabriela Kania [2] and Marijke Brink [1,*]

[1] Cardiobiology, Department of Biomedicine, University of Basel and University Hospital Basel,
4031 Basel, Switzerland; lifen.xu@unibas.ch (L.X.); philipp.klinger@me.com (P.K.); ch.morandi@unibas.ch (C.M.); lilia.lepine.fr@gmail.com (L.L.)

[2] Center of Experimental Rheumatology, Department of Rheumatology, University Hospital Zurich,
University of Zurich, 8091 Zurich, Switzerland; lukas.minder@gmx.ch (L.M.); gabriela.kania@uzh.ch (G.K.)

[3] Laboratory of Physiopharmacology, Faculty of Medicine and Health Sciences, Faculty of Pharmaceutical, Biomedical and Veterinary Sciences, Campus Drie Eiken, University of Antwerp, 2610 Antwerp, Belgium; pieter-jan.guns@uantwerpen.be (P.-J.G.); matthias.bosman1@gmail.com (M.B.)

[4] Laboratory of Tumor Heterogeneity, Metastasis and Resistance, Department of Biomedicine,
University of Basel, University Hospital Basel, 4031 Basel, Switzerland; m.coissieux@unibas.ch

[5] School of Cardiovascular and Metabolic Medicine and Sciences, MRC/BHF Centre of Research Excellence
in Advanced Cardiac Therapies, King's College London, London WC2R 2LS, UK; thierry.pedrazzini@kcl.ac.uk

* Correspondence: parisa.aghagolzadeh@unibas.ch (P.A.); marijke.brink@unibas.ch (M.B.)

Supplemental Figures

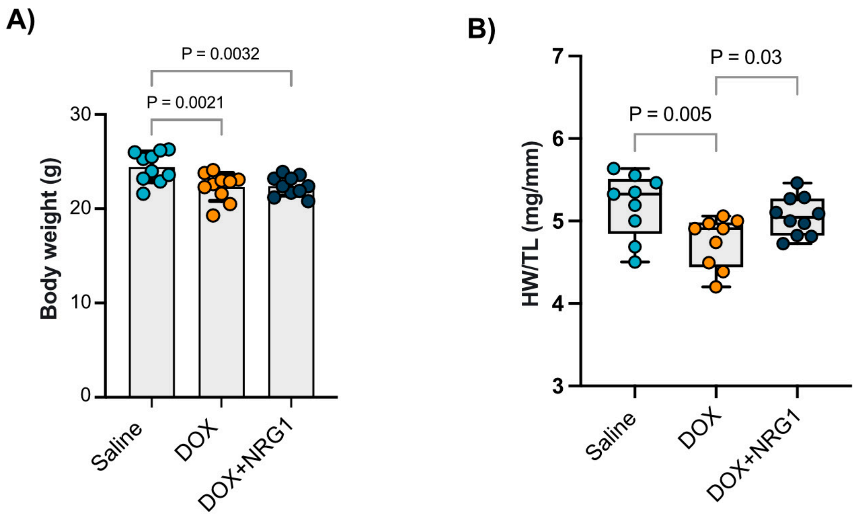

Suppl. Fig. S1. Effects of DOX and NRG1 on body weight and heart weight/tibia length ratio in tumor-bearing mice. A) Body weight at the end of the experiment. **B)** Heart weight to tibia length ratio (HW/TL) at sacrifice. N = 9–10 mice per group (biological replicates); each symbol represents one mouse. Data are mean ± SD. Statistical significance was assessed using one-way ANOVA followed by Fisher's LSD test when the overall ANOVA was significant; exact p-values are shown on the graphs.

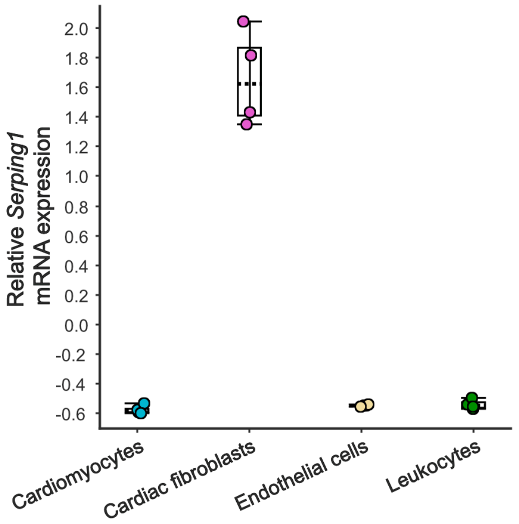

Suppl. Fig. S2. *Serping1* expression is enriched in adult cardiac fibroblasts. Relative *Serping1* mRNA expression in sorted adult mouse cardiac cell populations from the publicly available bulk RNA-seq dataset reported by Quaife-Ryan et al [1]. All cell populations shown are from adult mouse hearts. *Serping1* expression was enriched in adult cardiac fibroblasts compared with adult cardiomyocytes, endothelial cells, and leukocytes. Data are shown as individual biological replicates.

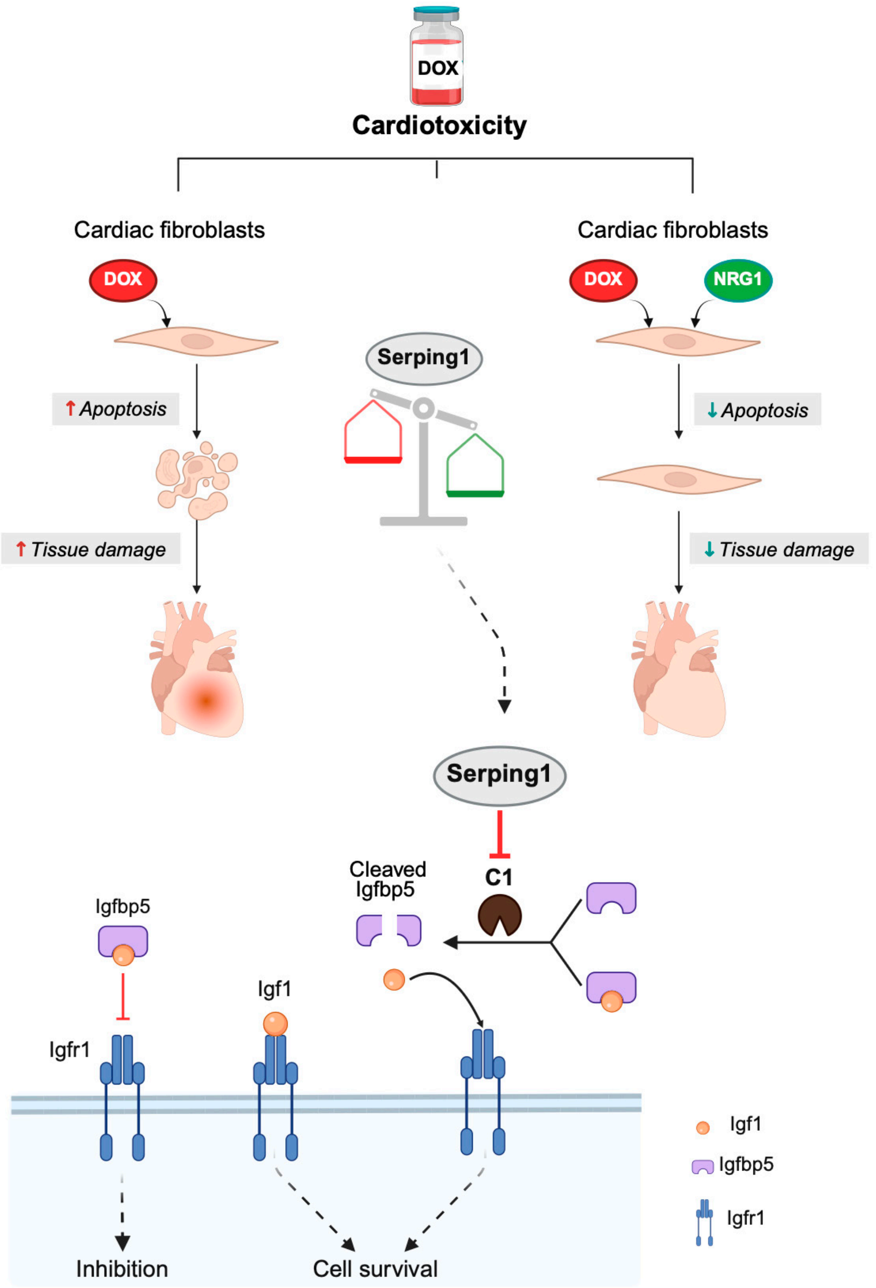

Suppl. Fig. S3. NRG1 downregulates Serping1, which modulates Igf1/Igfbp5 survival pathways in cardiac fibroblasts.
Schematic of the proposed mechanism: DOX elevates Serping1 in CFs, increasing apoptosis and tissue damage. NRG1 co-treatment lowers Serping1 and is cardioprotective. Mechanistically, Serping1 inhibits the complement protease C1; when Serping1 is high (DOX), C1 activity is restrained, Igfbp5 remains intact, and Igf1r signaling is reduced. When Serping1 is reduced (NRG1 or Serping1 siRNA), C1 cleaves Igfbp5, releasing Igf1 to activate Igf1r and enhance cell survival.

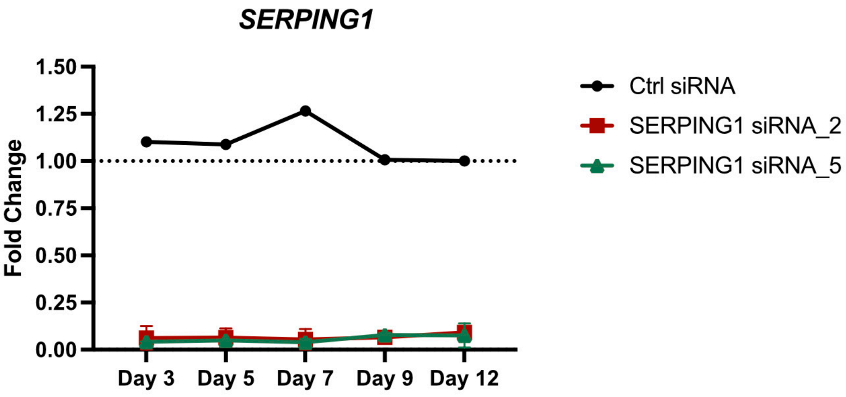

Suppl. Fig. S4 Efficient and stable knockdown of SERPING1 in human cardiac fibroblasts prior to microtissue assembly. Human cardiac fibroblasts were transfected with control siRNA or two independent SERPING1-targeting siRNAs (siRNA_2 and siRNA_5) and cultured for up to 12 days. SERPING1 transcript levels were measured by qPCR at the indicated time points and expressed as fold change relative to control siRNA (day 3).

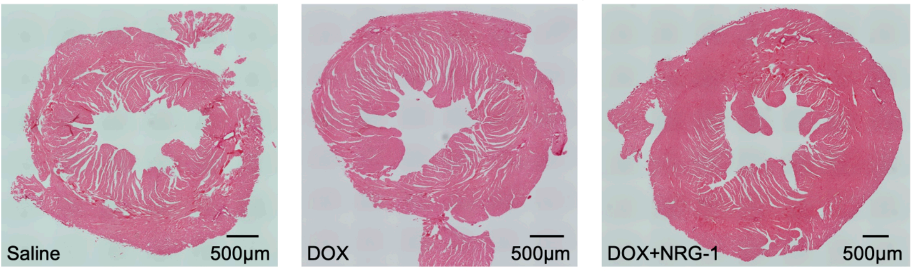

Suppl. Fig. S5. Picro-Sirius Red staining reveals no detectable interstitial fibrosis. Representative Picro-Sirius Red stained cross-sections of mouse hearts from the saline, DOX, and DOX + NRG1 treatment groups, collected at the same time point as in Figure 1. Scale bar = 500 µm. Quantitative analysis revealed no statistically significant differences in interstitial collagen deposition between treatment groups.

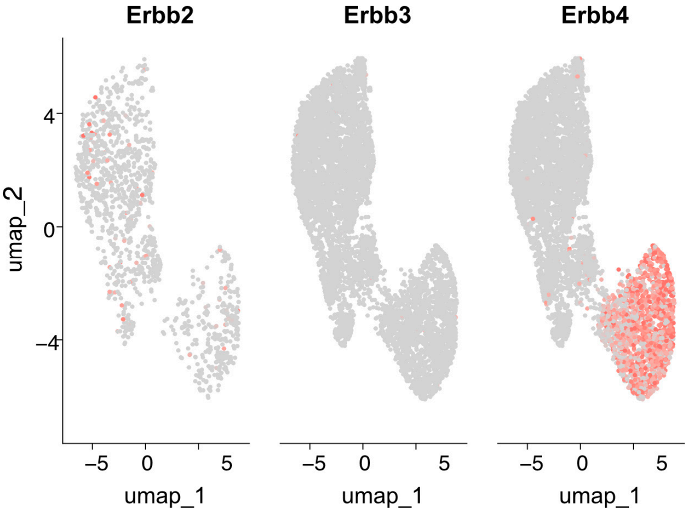

Suppl. Fig. S6. Feature plots of NRG1 receptor ErbB family members in cardiac fibroblasts. UMAP representations from single-cell RNA sequencing show the expression of Erbb2, Erbb3, and

Erbb4. Red indicates cells with detectable expression of the respective receptor, while gray represents non-expressing cells.

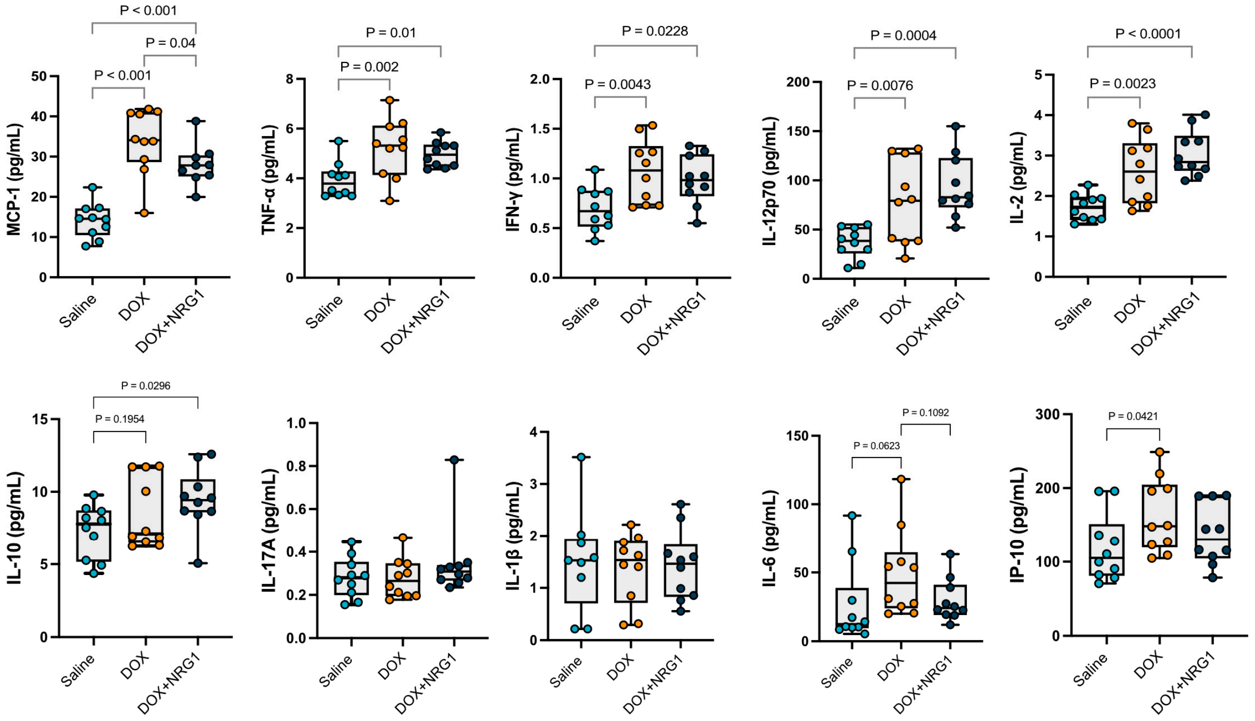

Suppl. Fig. S7. Serum cytokine and chemokine profiles in saline, DOX and DOX+NRG1-treated mice. Serum levels of MCP-1, TNF-α, IFN-γ, IL-12p70, IL-2, IL-10, IL-17A, IL-1β, IL-6 and IP-10 measured using the MSD U-PLEX ®Proinflammatory Combo 1 (Mouse) panel .N = 10 mice per group (biological replicates); each symbol represents one mouse. Data are mean ± SD. Group differences were analyzed by one-way ANOVA followed by Fisher's LSD test when the overall ANOVA was significant; exact p-values are shown on the graphs.

Supplementary table S3

Primer sequences (5' → 3')

Species	Gene	Forward primer	Reverse primer
Rat	*Gapdh*	GAT GGT GAA GGT CGG TGT GAA	TTGAACTTGCCGTGGGTAGAG
Rat	*Serping1*	GCGGAGAACACCAACCA	TTTCTTCCACTTGGCACTC
Rat	*Igfbp5*	CCTGCACCTGAGATGAGAC	GGCTTGCACTGCTTTCTC
Human	*SERPING1*	CCAGAGTCCTAAGCAACAACAG	GTCTTCCACTTGGCACTCAG

siRNA

Species	Name	Reference	Supplier
Rat	*Serping1* siRNA	Rn_RGD:735225_3 FlexiTube	Qiagen
Human	*SERPING1* siRNA	FlexiTube GeneSolution GS710	Qiagen

1. Quaife-Ryan GA, Sim CB, Ziemann M, Kaspi A, Rafehi H, Ramialison M, El-Osta A, Hudson JE, Porrello ER: **Multicellular Transcriptional Analysis of Mammalian Heart Regeneration**. *Circulation* 2017, **136**(12):1123-1139.
